# Epidemiology of *Tardiomyces blankii* (formerly *Candida blankii*) and related species: results of the International “Epiblankii” Study

**DOI:** 10.1128/jcm.00419-26

**Published:** 2026-09-30

**Authors:** Margot Paumier, Bram Spruijtenburg, Anne-Cécile Normand, Jacques F. Meis, Suhail Ahmad, Summiya Nizamuddin, Andrew M. Borman, Alireza Abdolrasouli, João Nobrega de Almeida Junior, Estelle Sabourin, Arnaud Riat, Françoise Botterel, Suefay Harumi Liu, Damien Costa, Grégoire Pasquier, Boualem Sendid, Renaud Piarroux, Theun de Groot, Eelco F. J. Meijer, Arnaud Fekkar

**Affiliations:** 1 AP-HP, Groupe Hospitalier La Pitié-Salpêtrière, Parasitologie Mycologie, Paris, France; 2 Radboudumc-CWZ Center of Expertise for Mycology, Nijmegen, the Netherlands; 3 Department of Medical Microbiology and Immunology, Canisius-Wilhelmina Hospital (CWZ)/Dicoon 6030 , Nijmegen, the Netherlands; 4 National Reference Centre "INuSuAle" (Digital Identification, Surveillance, Alert) (associated with the NRC for Invasive Mycoses and Antifungal), La Pitié-Salpêtrière Hospital, Paris, France; 5 Institute of Translational Research, Cologne Excellence Cluster on Cellular Stress Responses in Aging-Associated Diseases (CECAD), Excellence Center for Medical Mycology (ECMM), University of Cologne 14309, Cologne, Germany; 6 Department of Microbiology, Faculty of Medicine, Kuwait University 37603, Jabriya, Kuwait; 7 Department of Pathology, Shaukat Khanum Memorial Cancer Hospital and Research Centre 66799, Lahore, Pakistan; 8 UKHSA Mycology Reference Laboratory, Southmead Hospital 159003, Bristol, United Kingdom; 9 MRC Centre for Medical Mycology, University of Exeter 3286, Exeter, United Kingdom; 10 Laboratório Especial de Micologia, Disciplina de Infectologia, Universidade Federal de São Paulo 28105, São Paulo, Brazil; 11 Laboratoire de Parasitologie-Mycologie, CHU Necker-Enfants Malades 246596 , Paris, France; 12 Diagnostic Department, Laboratory of Bacteriology, University Hospital of Geneva, Geneva, Switzerland; 13 AP-HP, Groupe Hospitalier Henri Mondor, Parasitologie Mycologie, Créteil, France; 14 Vancouver Coastal Health, Division of Medical Microbiology and Infection Control 25469, Vancouver, British Columbia, Canada; 15 Rouen University Hospital, Laboratory of Parasitology-Mycology, Rouen, Normandie, France; 16 Laboratoire de Parasitologie-Mycologie, CHU de Montpellier, Université de Montpellier, CNRS, IRD, MiVEGEC 27037, Montpellier, France; 17 CHU Lille, Institut de Microbiologie, Service de Parasitologie-Mycologie, INSERM U1285, CNRS UMR 8576, Université de Lille 27023, Lille, France; 18 Sorbonne Université, CIMI-Paris, AP-HP, Service de Parasitologie-Mycologie, Hôpital de la Pitié-Salpêtrière 26933, Paris, France; University of Calgary, Calgary, Alberta, Canada

**Keywords:** *Tardiomyces *spp., antifungal resistance, MSI, mass spectrometry, emerging fungal pathogens

## Abstract

*Tardiomyces blankii* (previously known as *Candida blankii*) is an understudied emerging fungal pathogen with an unusual susceptibility profile. The aim of this study was to improve our understanding of the epidemiology of *Tardiomyces* spp. Cases were collected through two surveillance networks. The first was the Mass Spectrometry Identification (MSI) international network (La Pitié-Salpêtrière Hospital, Paris, France), based on mass spectrometry to identify fungal pathogens. The second was Canisius-Wilhelmina Hospital, Center of Expertise for Mycology (Nijmegen, the Netherlands). MSI members who had identified at least one *T. blankii* isolate between 2019 and 2025 collected retrospective data and provided their isolates. *In vitro* antifungal susceptibility testing (AFST) was carried out for 10 antifungal drugs. Whole-genome sequencing single-nucleotide polymorphism analysis was conducted to assess genetic diversity. Over the period 2019–2025, 251 spectra submitted to the MSI application were identified as *T. blankii sensu lato*. From the two surveillance networks, 46 isolates were available for further analysis, leading to the identification of 34 *T. blankii*, 11 *Tardiomyces depauwii*, and 1 *Tardiomyces digboiensis*. Clinical isolates were related to 29 cases of infection/colonization. The AFST data showed high minimum inhibitory concentration (MIC) values for azoles and echinocandins. Interestingly, these isolates demonstrated elevated MICs for anidulafungin and caspofungin but not micafungin. A low genetic diversity was found in *T. depauwii* isolates, while *T. blankii* isolates showed considerable genetic variation, although most French isolates formed a distinct clade. Our study sheds light on these rare human-pathogenic yeast species that exhibit reduced susceptibility to antifungal drugs.

**IMPORTANCE:**

*Tardiomyces* spp. (formerly classified as *Candida blankii*) are emerging opportunistic yeasts that remain poorly recognized in routine clinical microbiology. To improve our understanding of the epidemiology of *Tardiomyces* spp., we combined data from the Mass Spectrometry Identification international network (Paris, France) and the Center of Expertise for Mycology (Nijmegen, the Netherlands) with whole-genome sequencing and antifungal susceptibility testing. We show that improving MALDI-TOF MS databases markedly enhances species identification, allowing discrimination between *Tardiomyces blankii*, *Tardiomyces depauwii*, and *Tardiomyces digboiensis*. Our findings also identify at-risk patients, describe the antifungal susceptibility profiles of these emerging pathogens, and reveal distinct patterns of genetic diversity. As uncommon fungal pathogens are increasingly encountered in vulnerable patients, accurate laboratory identification is essential for surveillance, epidemiology, and clinical management. These results provide practical tools and new knowledge that will facilitate the recognition and monitoring of this neglected genus.

## INTRODUCTION

Invasive fungal infections caused by yeasts are a threat to many of the most vulnerable hospitalized patients (1, 2). Most infections are caused by yeasts of the *Candida* genus, and within this genus, most are part of the World Health Organization (WHO) fungal priority pathogen group, including *Candida albicans*, *Candida parapsilosis*, *Nakaseomyces glabratus* (syn. *Candida glabrata*), *Candida auris*, and *Candida tropicalis*. However, many other genera and species may also be involved, albeit less frequently. Among the rarely involved species, one can distinguish between those that have been described for a long time and are frequently found in the human environment but have limited pathogenicity (e.g., *Saccharomyces cerevisiae*) versus those that can be defined as having an ecological niche that makes their incidence in human infections very low. The medical community pays particular attention to the latter group because they may possess one or more of the following characteristics: an emerging nature, the ability to spread clonally, and a propensity for multiresistance to antifungal agents (3).

In recent years, invasive infections due to less common fungal species have been increasingly reported (4). There are many different reasons that can be put forward to explain this phenomenon, including not only global climate change and an increase in the number of vulnerable patients but also advances in diagnostic approaches and accurate identification capacities of fungi in clinical settings. The ongoing evolution of yeast taxonomy is also contributing to the characterization of emerging pathogens. One specific example of this is the yeast *Tardiomyces blankii* (previously known as *Candida blankii*). This specific yeast was first described in Canada in 1968 by Buckley and van Uden (5). Its pathogenicity has remained virtually unknown since then, but it has attracted increasing interest since the 2010s, with several published case reports and descriptions of a hospital outbreak (6–8). The genus *Tardiomyces* was recently described based on whole-genome sequencing (WGS) analyses, which placed *C. blankii* in a distinct branch in the order *Dipodascales* (9, 10). Among 26 (17 clinical and nine environmental) isolates previously identified as *C. blankii*, a new species was recognized, which ultimately led to the erection of a new genus that included *T. blankii*, *Tardiomyces digboiensis*, and *Tardiomyces depauwii*. The ability of this neglected lineage to cause nosocomial transmission, combined with the acquisition of resistance to antifungal agents, might complicate therapeutic management of patients and deserves particular attention.

In the context of describing new emerging fungal species, MALDI-TOF mass spectrometry identification has become a common tool in many microbiology laboratories, as it enables rapid and accurate identification of fungal pathogens. Its usefulness is undisputed, provided that the databases are regularly updated and implemented with the spectra of rare/newly described species. Mass Spectrometry Identification (MSI, accessed at https://msi.happy-dev.fr/) is a free, online application developed and hosted at the Sorbonne University and La Pitié-Salpêtrière Hospital, Paris, France, which permits the identification of a large number of fungal agents from their MALDI-TOF mass spectra (11). Moreover, it gives users access to a network of hundreds of others and makes it easier to retrieve and share information, particularly regarding rare species. We conducted a retrospective and prospective multicenter study called “Epiblankii” to examine the epidemiology of *T. blankii* and related species *T. depauwii* and *T. digboiensis*, including a review of the species distribution, genetic diversity, patient profile, and antifungal susceptibility. Concurrently, we sought to improve the MSI database by incorporating these new isolates into the database.

## MATERIALS AND METHODS

### Isolates and clinical data collection

We contacted members of the MSI network whose activity indicates at least one identification of a *T. blankii* isolate between 2019 (the year the MSI application was launched) and 2025, in France and abroad. Isolates available at Canisius-Wilhelmina Hospital (CWZ; Nijmegen, the Netherlands) were also shared and sent to the La Pitié-Salpêtrière Hospital (Paris, France). Patients were classified as colonization when isolates were retrieved from non-sterile sites and defined as invasive infection when they were isolated from blood.

### Mass spectrometry analysis

All isolates were analyzed by MALDI-TOF mass spectrometry (Bruker Microflex). New reference spectra were obtained from cultures grown for 48 hours on various media, including blood agar, chocolate agar, Sabouraud agar, malt agar, and chromogenic agar (CHROMagar *Candida*). Two protein extraction methods were employed to create the reference spectra: a full extraction protocol using ethanol, formic acid, and acetonitrile as previously described, and a direct on-plate extraction using formic acid alone (11). All extracts were overlaid with alpha-cyano-4-hydroxycinnamic acid matrix before acquisition on a Microflex instrument (Bruker).

### Determination of minimum inhibitory concentration

*In vitro* antifungal susceptibility testing was carried out according to the European Committee on Antimicrobial Susceptibility Testing (EUCAST) method for 10 antifungal agents: fluconazole, voriconazole, posaconazole, isavuconazole, itraconazole, micafungin, anidulafungin, caspofungin, amphotericin B, and 5-fluorocytosine (5-FC). *Candida parapsilosis* ATCC 22019 was used as the control strain. Minimum inhibitory concentrations (MICs) were read after 48 hours of incubation at 37°C. The tested concentrations ranged from 0.125 to 64 mg/L for fluconazole and 5-FC and from 0.008 to 4 mg/L for the other antifungals.

### WGS single-nucleotide polymorphism analysis

DNA of the isolates was extracted and subsequently purified using the MagNA Pure 96 system according to the manufacturer’s instructions as described previously (9). Final DNA concentration was measured with the Qubit 3.0 Fluorometer (Thermo Fisher Scientific, Waltham, MA, USA). Genomic libraries were prepared and sequenced with the Illumina Novaseq 600 platform (Illumina, San Diego, CA, USA) and two times 150 bp paired-end-read mode. Generated reads were aligned to the *Tardiomyces blankii* NRRL Y-17068 reference genome (GCA_030573285.1), and single-nucleotide polymorphism (SNP) analysis was executed as previously described (12). In short, with BWA v0.7.19, reads were aligned to the reference genome, and resulting BAM files were filtered. SNPs were called using Freebayes v1.3.9, and the inferred phylogenetic relatedness was visualized with MEGA11 v11.0.10 and iTOL v6. Previously, this SNP calling pipeline was validated with a *C. auris* benchmark data set. Isolates previously sequenced were included as control isolates (Table S1) (13). Raw read data generated during the current study have been submitted to the National Center for Biotechnology Information Sequence Read Archive under BioProject IDs PRJNA1302418 and PRJNA1302739. Raw reads are available under IDs SRR34885843-SRR34885857 and SRR-34901539-SRR34901552. BioSample IDs are available under SAMN50477854-SAMN50477868 and SAMN50490454-SAMN50490467.

## RESULTS

### MSI/INuSuAle surveillance network data

**Fig 1:**
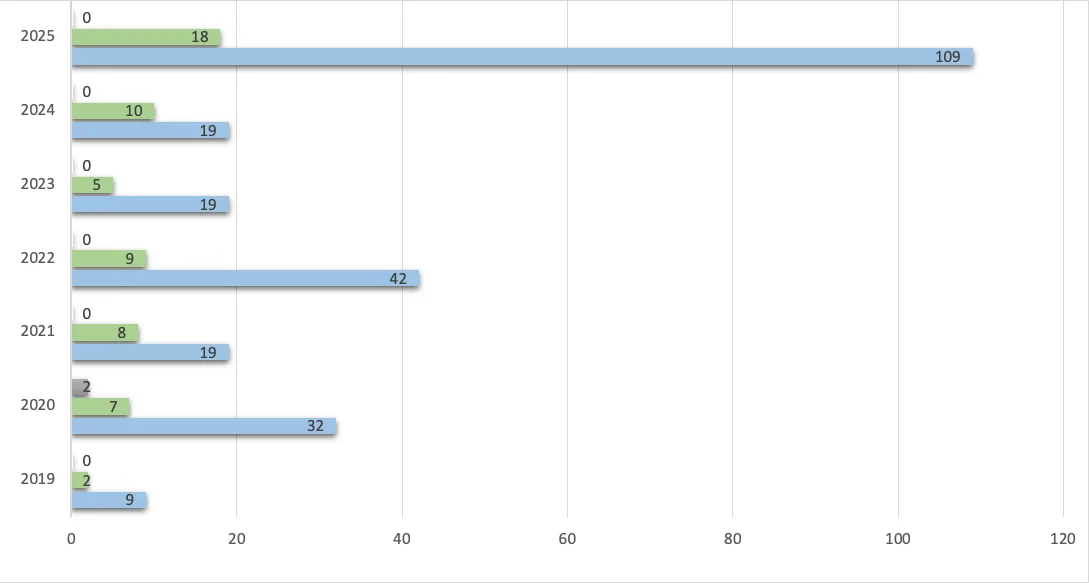
Number of centers that submitted at least one spectrum identified as *Candida blankii*/*Tardiomyces* spp. to the MSI application between 2019 and 2025 (in green), the total number of spectra identified as *Tardiomyces* spp. after implementation of the MSI database with the three different species by all users of the application (in blue), and unidentified spectra after implementation of the MSI database (in gray).

The number of spectra and centers that enabled identification of *T. blankii* using the MSI network between 2019 and 2025 is shown in Fig. 1. The total number of *T. blankii* spectra over the period 2019–2025 was 251, which corresponded to 63 isolates. The number of spectra generated was relatively stable between 2019 and 2024 but significantly higher for the year 2025. We contacted 21 MSI users from several countries (Belgium, Canada, Costa Rica, Denmark, France, Germany, Italy, the Netherlands, Spain, Switzerland, Sweden, United States, United Kingdom, and Norway) for a more in-depth study of the *T. blankii* isolates they had identified. Finally, among 21 MSI users, 19 isolates were received from four countries and submitted for further analysis. The various collaborating centers are located in different cities in France (Paris, Montpellier, Rouen, and Lille), Belgium (Brussels), Canada (Vancouver), and Switzerland (Geneva).

### Mass spectrometry database improvement and isolate recharacterization

Twenty-seven WGS-characterized isolates from the CWZ collection were used to supplement the MSI database. Thus, 69 spectra of *T. depauwii* were created, along with 10 spectra of *T. digboiensis*. This permitted the redefinition of the spectral identifications previously obtained and allowed us to recharacterize the available isolates previously identified as *Candida blankii*. After inclusion in the database, a total of 251 spectra with an identification score above 20 by MSI (i.e., the cutoff considered for a relevant species-level identification) were considered. Reprocessing of the 251 spectra initially identified as *C. blankii* resulted in the reclassification of 134 spectra as *T. blankii* (28 isolates), 113 as *T. depauwii* (35 isolates), and 2 as *T. digboiensis*, while 2 were unidentified (Fig. 1). Thus, the MSI database enabled species identification in 99% of cases (249/251 spectra). In contrast, for *T. blankii*, out of 56 spectra analyzed using the Bruker database, only 39 could be correctly identified (70%). For *T. depauwii*, of the 84 spectra analyzed using the Bruker research use only (RUO) database, none could be identified (0%).

### Characteristics of clinical isolates

**Fig 2:**
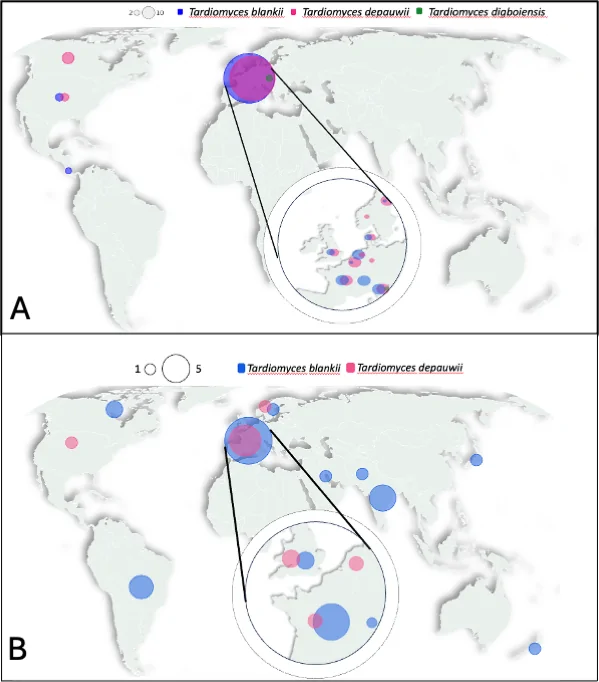
Geographical distribution of *Tardiomyces* spectra submitted to the MSI application, resulting in the identification of *Tardiomyces* spp. between 2019 and 2025 (**A**) and the geographical origin of *Tardiomyces* spp. isolates analyzed in the study (**B**). The map was created using the Flourish application (https://app.flourish.studio).

A total of 46 isolates were analyzed: 27 isolates from the CWZ collection that had previously been analyzed by WGS and 19 isolates collected via the MSI network. Following the improvement of the database, these 19 isolates were reidentified using the implemented version of MSI, and identification was confirmed using a molecular approach at CWZ. Of the 46 isolates, 34 were *T. blankii*; 11 were *T. depauwii*; and 1 was *T. digboiensis*, giving a total of 29 cases of infection/colonization. Additional data were available for 39 of the 46 isolates (29 *T. blankii*, 9 *T. depauwii*, and 1 *T. digboiensis*) from a total of 23 patients. Of the 29 *T. blankii* isolates, 23 were of human origin (79%); 4 were of environmental origin (14%); and 2 were of animal origin (7%). All of the *T. depauwii* isolates received were of human origin. The only *T. digboiensis* isolate was of environmental origin. In our study, we report nine cases of invasive infections and five cases of colonization caused by *T. blankii*. For *T. depauwii*, we describe a total of nine cases: five cases of invasive infections and four cases of colonization. The clinical characteristics of invasive infections for *T. blankii* and *T. depauwii* are shown in Table 1. Cases of colonization are also reported in three patients with respiratory conditions (two with cystic fibrosis and one with bronchiectasis). The geographical origin of the *T. blankii* and *T. depauwii* isolates sent by the various collaborating centers is described in Fig. 2.

**TABLE 1:** Clinical characteristics of 14 patients with *Tardiomyces* spp. invasive infections^*a*^

Clinical characteristics of patients with *Tardiomyces* spp. invasive infection	*Tardiomyces blankii*	*Tardiomyces depauwii*
Invasive infection	9	5
Invasive infection type		
Candidemia	8 (88.9)	5 (100)
Other	1 (11.1) (metacarpophalangeal osteoarticular infection)	0 (0)
Hospital unit		
Intensive care unit	6 (67)	0 (0)
General ward	1 (11)	1 (20)
Not determined	2 (22)	4 (80)
Comorbidities		
Prematurity	6 (67)	0 (0)
Chronic granulomatous disease	1 (11)	0 (0)
Cystic fibrosis	1 (11)	0 (0)
None	1 (11)	5 (100)
Immunosuppression		
Corticoids	1 (11)	0 (0)
Solid organ transplantation	0 (0)	1 (20)
None	7 (78)	4 (80)
Not available	1 (11)	0 (0)
Antifungal therapeutic		
Fluconazole	5 (55)	0 (0)
Amphotericin B	2 (23)	0 (0)
Voriconazole	1 (11)	0 (0)
Caspofungin	0 (0)	1 (20)
Not available	1 (11)	4 (80)

^*a*^ Data are expressed as numbers, with percentages in parentheses.

### Antifungal susceptibility testing

MICs were determined for 29 isolates in triplicate (during three separate experiments) and reported in 29 cases of infection/colonization caused by *T. blankii* and *T. depauwii* (Table 2). For *T. blankii*, MICs were high for all azoles. Regarding echinocandins, MICs appear to be lower for micafungin than for the other echinocandins for the two species, with lower geometric means for *T. depauwii* with a range from 0.031 to 0.25 mg/L. For amphotericin B, the MICs were lower than for azoles and echinocandins for the two species. This is also the case for flucytosine. We also compared the MICs found in this study with those already reported in the literature with alternative testing methods (CLSI and gradient concentration strip) (Table S2).

**TABLE 2:** Minimum inhibitory concentrations (MICs) of 10 antifungal agents reported in 29 cases of infection/colonization caused by Tardiomyces blankii and Tardiomyces depauwii determined by the EUCAST reference method^*c*^

Species (number of isolates)	Antifungal (mg/L)					<0.125	0.125	0.25	0.5	1	2	4	8	16	32	64	>64^*b*^	Geometric mean
Tardiomyces blankii (18)	FLZ					0	0	0	0	0	0	0	1	0	5	7	5	57.02
	5FC					0	14	4	0	0	0	0	0	0	0	0	0	0.15
	mg/L	<0.008	0.008	0.016	0.031	0.0625	0.125	0.25	0.5	1	2	4	>4^*a*^					
	VCZ	0	0	0	0	0	0	0	1	2	11	2	2					2.16
	PSZ	0	0	0	0	0	0	0	1	2	8	7	0					2.25
	ITZ	0	0	0	0	0	0	4	0	5	8	1	0					1.08
	ICZ	0	0	0	0	0	2	2	1	8	5	0	0					0.79
	AFG	0	0	0	0	1	0	0	2	3	2	4	6					2.33
	CAS	0	0	0	0	0	0	0	1	0	0	0	17					6.85
	MFG	0	0	0	0	0	3	2	1	1	0	0	11					2.08
	AMB	0	0	0	0	0	0	0	8	10	0	0	0					0.73
Tardiomyces depauwii (11)	mg/L					< 0,125	0.125	0.25	0.5	1	2	4	8	16	32	64	>64^*b*^	
	FLZ					0	0	0	0	0	0	0	0	0	0	0	11	128
	5FC					0	1	2	4	1	1	2	0	0	0	0	0	0.69
	mg/L	<0.008	0.008	0.016	0.031	0.0625	0.125	0.25	0.5	1	2	4	>4^*a*^					
	VCZ	0	0	0	0	0	0	0	0	0	0	0	11					8
	PSZ	0	0	0	0	0	0	0	0	2	5	4	0					2.27
	ITZ	0	0	0	0	0	0	0	0	5	2	1	3					2
	ICZ	0	0	0	0	0	0	0	0	2	3	4	2					2.92
	AFG	0	0	0	0	0	0	1	1	2	3	0	4					2.13
	CAS	0	0	0	0	0	0	0	2	1	0	0	8					4
	MFG	0	0	0	1	0	2	8	0	0	0	0	0					0.18
	AMB	0	0	0	0	0	0	0	10	1	0	0	0					0.53

^*a*^ Isolates with MIC >4 mg/L were considered to be 8 mg/L.

^*b*^ Isolates with MIC >64 mg/L were considered to be 128 mg/L.

^*c*^ AFG, anidulafungin; AMB, amphotericin B; CAS, caspofungin; 5FC, 5-fluorocytosin; FLZ, fluconazole; ICZ, isavuconazole; ITZ, itraconazole; MFG, micafungin; PSZ, posaconazole; VCZ, voriconazole.

### Nosocomial transmission investigation

**Fig 3:**
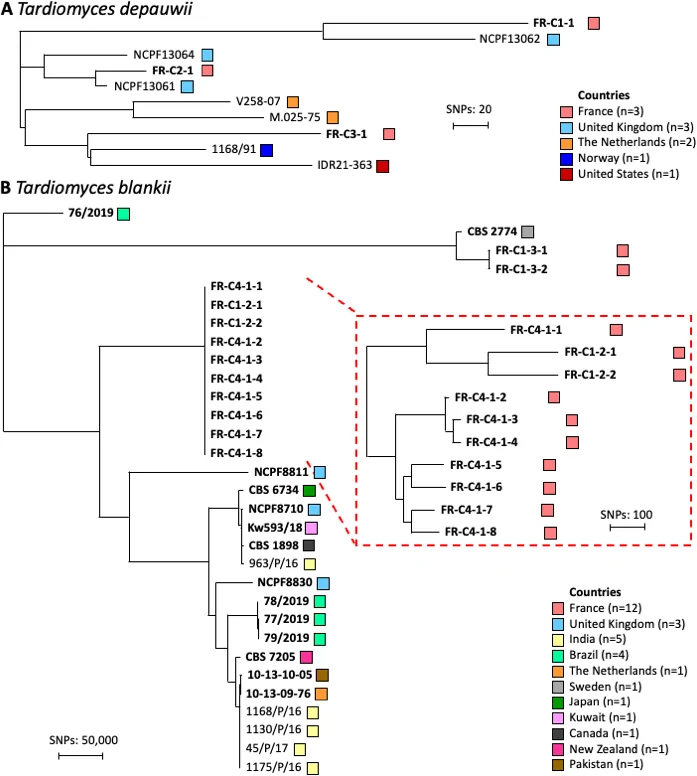
Phylogenetic tree of *Tardiomyces depauwii* (**A**) and *Tardiomyces blankii* (**B**) based on single-nucleotide polymorphisms (SNPs). Novel isolates sequenced in this study are indicated in bold.

For 3 novel *T. depauwii* isolates and 26 novel *T. blankii* isolates, Illumina sequencing was performed (Table S3). A WGS SNP analysis was conducted on three novel *T. depauwii* isolates, while seven previously sequenced isolates were included as controls. A low genetic diversity was found, with differences between isolates ranging from 38 to 501 SNPs (Fig. 3A). The French *T. depauwii* isolates did not group together and were most closely related to a British isolate. In contrast, the 31 *T. blankii* isolates (26 novel isolates, with 5 Indian strains included as controls) showed high genetic variation with up to ~900,000 SNPs (Fig. 3B). Notably, French isolates clustered into two groups, with one cluster consisting of two isolates (FR-C1-3-1 and FR-C1-3-2) differing by 936 SNPs that originated from Paris. Most closely related to this cluster was the Swedish environmental isolate CBS 2774, differing by at least 48,731 SNPs. The other cluster consisted of the remaining 10 isolates and differed by 79–823 SNPs and was not closely related to the first French cluster. In addition, three environmental Brazilian isolates (77/2019, 78/2019, and 79/2019) clustered as a separate group and differed by 1,891 SNPs at most. Moreover, four Indian outbreak isolates (1130/*P*/, 1168/*P*/16, 1175/*P*/16, and 45/*P*/17) clustered with Dutch (10–13) and Pakistani (10–13) clinical isolates, with a maximum difference of 2,157 SNPs.

## DISCUSSION

The emergence of new fungal pathogens is a matter of medical concern, especially when they have the potential to become resistant to antifungals or cause outbreaks (14). This article focused on the newly described genus *Tardiomyces*, which is still poorly described but whose emerging potential warrants attention. This study enables us to determine some patient clinical profiles and isolate susceptibilities for the species formerly identified as *C. blankii*, now recharacterized as *Tardiomyces blankii*, *Tardiomyces depauwii*, and the distinct species *Tardiomyces digboiensis*. This new genus has been proposed because of the significant phylogenetic distance between these three species and *Candida* (9).

*C. blankii* was first described in the 1960s by Dr. Blank and then characterized in 1968 by Buckley and van Uden (5). This yeast has rarely been identified in cases of invasive infections: a case of fungemia described in a young girl with cystic fibrosis who had undergone a lung transplant in Brazil (15), a fungemia case in a 27-week-old premature baby in Kuwait in 2018 (7), an osteoarticular infection in a patient with chronic granulomatous disease in France in 2024 (8), and more recently, the description of 126 *T. blankii* bloodstream infections among neonates in an intensive care unit in Bangladesh (16). This yeast has also been described in the United States in two case reports: in an immunocompetent patient with possible endocarditis and in a case of fungemia in a patient with severe COVID-19 (17, 18). It gained significant medical interest following the description of an outbreak in India, with nine cases of fungemia in newborns (6). Cases of colonization have also been described, notably in a cystic fibrosis patient in Argentina (19) and on the hands of a 2-month-old infant in Russia suffering from atopic dermatitis (20).

However, the number of cases of colonization and infection reported in the literature remains limited to date. Knowledge may be limited by the challenges in reliable identification, as underscored by a 2025 study describing two cases that highlighted the difficulty of *Candida blankii* identification using commercial mass spectrometry databases (21). Our study found similar results, since the Bruker RUO database did not allow identification of *T. depauwii* and correctly identified *T. blankii* in only 70% of cases. On the other hand, the supplemented MSI database with 283 reference spectra allows species identification in 99% of cases and shows that the addition of spectra from isolates confirmed by WGS is essential. Interestingly, of the 19 isolates received during the study, only 4 (21%) were reclassified as *T. depauwii*. This percentage is slightly lower, given that 45% of the spectra previously identified as *C. blankii* since 2019 were reclassified as *T. depauwii* after the implementation of the MSI database. Finally, it should be noted that the Vitek MS database from BioMérieux contains a reference for identifying *T. blankii*, but we did not perform a comparison using a BioMérieux device.

Although the majority of isolates were of human origin, some were of environmental origin (5/46), in line with what was previously reported by Pham et al. and Sandhu et al., with *T. blankii* detected on Vietnamese coasts and in flower nectar in India (22, 23).

We observed that *T. blankii* was responsible for infections or colonization in patients with specific underlying diseases, namely, prematurity (*n* = 6) and diseases related to pulmonary inflammation, such as cystic fibrosis (*n* = 3), bronchiectasis (*n* = 1), and chronic granulomatous disease (*n* = 1). Interestingly, these patients' risk factors have one common feature: they are characterized by increased production of polyamines such as putrescine (24–26). A distinct feature of *T. blankii* is its ability to use polyamines as a source of nutrition (27). This is also true for other yeasts in the *Dipodascales* order, namely, *Stephanoascus ciferrii* (also known as *Trichomonascus ciferrii* or formerly *Candida ciferrii*), which is found in patients with similar clinical backgrounds. Finally, it should be noted that *T. blankii* can be misidentified as *S. ciferrii* using identification platforms or mass spectrometry with insufficient databases (7). Thus, the ability of these two closely related species to colonize/infect these types of patients could be linked in part to their specific metabolic capabilities.

Despite the small number of patients, for both species, the majority of cases were invasive infections (64% for *T. blankii* and 56% for *T. depauwii*). Some authors point to the potential of this species to cause nosocomial outbreaks, with clonal isolates showing reduced susceptibility to antifungal agents. This was the case in nine premature infants in India. In contrast, our study did not indicate clonal transmission among isolates from invasive infections.

Although there are no clinical breakpoints or ECOFFs associated with these species, the MICs found in our study were consistently elevated for most azoles and echinocandins when compared to common yeasts, suggesting intrinsic resistance mechanisms for the genus *Tardiomyces*. When comparing the MICs obtained for echinocandins with those from previous studies (Table S2), considerable variation is observed for *T. blankii*. The MICs for micafungin are generally lower than those reported in this study, which is in concordance with a previous study that investigated genetically distinct strains (9). Most reports focused on a single isolate, but the study by Chowdhary et al. examined nine isolates with a mean MIC for micafungin of 0.06 mg/L; however, this involved a clonal outbreak among nine newborns. Regarding caspofungin, it should be noted that EUCAST and CLSI guidelines do not recommend testing this drug due to high interlaboratory variability (28). MICs reported here with the azole antifungal agents are higher than those previously described. This may likely be explained by the different methodologies. The MICs for azoles in *T. depauwii* appear to be higher than in *T. blankii*, as previously reported by Spruijtenburg et al. (9). In previously published case reports of invasive infections, the use of amphotericin B, fluconazole, voriconazole, itraconazole, and micafungin gave a satisfactory outcome. It appears that despite high MICs, fluconazole was used in the vast majority of cases of fungemia, where patients survived. Hence, we suggest caution when comparing MICs to yeast species with defined breakpoints, as the *in vivo* correlation with *in vitro* results may be discordant. These observations suggest that current MIC values may not reliably predict clinical outcomes for *Tardiomyces* spp., underscoring the need for pharmacodynamic and outcome-based studies. MIC values obtained from clinical isolates were not notably different from environmental isolates.

Regarding the outbreak investigation, the WGS SNP analysis of three novel *T. depauwii* isolates showed that isolates were closely related, as previously reported. This indicates a single introduction of *T. depauwii* into the human population, which is to date geographically restricted to North and Western Europe and New York, USA. As this species can easily be misidentified as *T. blankii*, WGS should be performed on *T. blankii* or *T. depauwii* isolates from outside Europe to confirm a single introduction or uncover novel lineages. Moreover, environmental sampling or metagenomics sequencing could uncover if more lineages are still present or have become extinct. Conversely, for *T. blankii*, a high number of SNPs were found with several clusters and singletons, indicating few bottlenecks and a balanced evolution for this species. Surprisingly, all French isolates segregated into two clusters, indicating a recent shared ancestor and limited geographical dissemination for both groups. Given that isolates differed at least by 79 SNPs, patients likely acquired the yeast from an environmental source, with nosocomial transmission being less likely. To date, *T. blankii* has been isolated from diverse environmental niches, like sand, sugarcane, animals, and soil, making it challenging to identify the environmental niche of the two French clades. Last, the high genetic diversity in clinical samples showed that isolates with diverse genetic backgrounds can cause infections. Inclusion of more isolates with clinical information may indicate if genotypes display different virulence.

Our study has a few limitations: the number of isolates available for further analysis was limited compared to the number of MALDI-TOF identifications, and the amount of clinical information compiled for invasive infections is also limited. Furthermore, the sample origin of cultures remained unknown for some isolates.

### Conclusion

*Tardiomyces* spp. have a specific antifungal susceptibility profile and can colonize/infect particular groups of patients. The use of mass spectrometry coupled with the MSI application makes it possible to correctly identify these species, which is important, given their emerging potential. MICs found in our study were consistently elevated for most azoles and echinocandins when compared to common yeasts, suggesting intrinsic resistance mechanisms for the genus *Tardiomyces*. WGS indicated a low genetic diversity for *T. depauwii* and French *T. blankii* strains, although no correlation was found between the genetic background and the antifungal susceptibility profile.

## SUPPLEMENTAL MATERIAL

Table S1 jcm.00419-26-s0001.pdf Overview of *Tardiomyces* spp. control isolates extracted from the NCBI SRA database.

Table S2 jcm.00419-26-s0002.pdf Comparison of previously reported minimum inhibitory concentration (MICs) values for 10 antifungal drugs for *Tardiomyces* spp. (formerly Candida blankii) isolates

Table S3 jcm.00419-26-s0003.pdf Whole genome sequencing metrics of 3 Tardiomyces depauwii and 25 Tardiomyces blankii isolates.

ASM does not own the copyrights to Supplemental Material that may be linked to, or accessed through, an article. The authors have granted ASM a non-exclusive, world-wide license to publish the Supplemental Material files. Please contact the corresponding author directly for reuse.
