# Supplementary material for: Epidemiology of Tardiomyces blankii (formerly Candida blankii) and related species: results of the International “Epiblankii” Study
Source: J Clin Microbiol. 2026 Sep 30;64(11):e00419-26. doi: 10.1128/jcm.00419-26 (PMC13753830; doi:10.1128/jcm.00419-26)
Supplement: Supplementary file 1 [file jcm.00419-26-s0001.pdf]

ID	Species	SRA	Biosample ID
963/P/16	*T. blankii*	SRR10023786	SAMN11653514
1130/P/16	*T. blankii*	SRR10023777	SAMN11653526
1168/P/16	*T. blankii*	SRR10023885	SAMN11653548
1175/P/16	*T. blankii*	SRR10023892	SAMN11653550
45/P/17	*T. blankii*	SRR10024079	SAMN11653663
IDR21-363	*T. depauwii*	SRR26562494	SAMN38035623
NCPF13064	*T. depauwii*	SRR25761586	SAMN37150274
NCPF13062	*T. depauwii*	SRR25761587	SAMN37150273
NCPF13061	*T. depauwii*	SRR25761588	SAMN37150272
10-13-19-96	*T. depauwii*	SRR25761589	SAMN37150271
10-13-19-95	*T. depauwii*	SRR25761590	SAMN37150270
1168/91	*T. depauwii*	SRR25761591	SAMN37150269

Supplemental Data Table S1: Overview of *Tardiomyces* spp. control isolates extracted from the NCBI SRA database.
