# Supplementary material for: Epidemiology of Tardiomyces blankii (formerly Candida blankii) and related species: results of the International “Epiblankii” Study
Source: J Clin Microbiol. 2026 Sep 30;64(11):e00419-26. doi: 10.1128/jcm.00419-26 (PMC13753830; doi:10.1128/jcm.00419-26)
Supplement: Supplementary file 2 [file jcm.00419-26-s0002.pdf]

				Mean MIC (mg/liter), GM[a]									
**Organisms**	**Reference**	**Methods**	**No. of isolates**	**ITZ**	**VCZ**	**PSZ**	**FLZ**	**ICZ**	**AFG**	**CAS**	**MFG**	**5FC**	**AMB**
Tardiomyces blankii	Zaragoza S. et *al.*	CLSI	1	<0.13	<0.13	<0.13	<0.13	<0.13	<0.13	<0.13	<0.13	<0.13	<0.13
	Al-Haqqan et *al.*	Gradient concentration strip	1	0.75	0.38	0.75	16.00	NA	0.19	0.25	0.13	0.03	0.13
	Nobrega de Almeda et *al.*	EUCAST	1	NA	0.50	NA	16.00	NA	0.35	NA	0.30		0.42
	Chowdhary et *al.*	CLSI	9	0.18	0.25	0.13	8.00	0.07	2.00	NA	0.06	NA	NA
	Kollu VS et *al.*	CLSI	1	0.50	0.25	1.00	16.00	NA	0.25	1.00	0.12	0.06	0.50
	Mirchin R. et *al.*	CLSI	1	1.00	8.00	2.00	256.00	NA	NA	0.25	0.50	NA	0.06
	Spruijtenburg B. et *al.*	CLSI	4	0.35	0.59	0.50	19.03	0.18	1.68	NA	0.30	NA	0.30
	Sabourin et *al.*	EUCAST	1	NA	0.50	0.50	8.00	0.25	NA	0.06	0.13	0.13	0.50
	This study	EUCAST	34	1.18	2.17	2.72	65.32	0.96	2.55	6.52	1.30	0.15	0.72
Tardiomyces depauwii	Spruijtenburg B. et *al.*	CLSI	7	0.30	5.38	0.45	128.00[b]	0.50	2.00	NA	0.28	NA	0.34
	This study	EUCAST	11	2.00	8.00	2.27	128.00	2.92	2.13	4.00	0.18	0.69	0.53
Tardiomyces digboiensis	Spruijtenburg B. et *al.*	CLSI	1	0.03	0.13	0.03	4.00	0.06	2.00	NA	2.00	NA	0.50
	This study	EUCAST	1	0.25	2.00	1.00	64.00	0.50	8.00	8.00	8.00	0.25	0.50

Supplemental Data Table S2: Comparison of previously reported Minimal Inhibitory Concentration (MICs) values for ten antifungal drugs for *Tardiomyces* spp. (formerly *Candida blankii*) isolates

Amphotericin B (AMB), itraconazole (ITZ), voriconazole (VCZ), posaconazole (PSZ), isavuconazole (ICZ), fluconazole (FLZ), anidulafungin (AFG), caspofungin (CAS), micafungin (MFG) and 5-fluorocytosin (5FC).
Not applicable (NA)
[a] GM, Geometric mean
[f] for 7 isolates, MICs above 64 were considered to be 128 mg/L
