# Supplementary material for: Epidemiology of Tardiomyces blankii (formerly Candida blankii) and related species: results of the International “Epiblankii” Study
Source: J Clin Microbiol. 2026 Sep 30;64(11):e00419-26. doi: 10.1128/jcm.00419-26 (PMC13753830; doi:10.1128/jcm.00419-26)
Supplement: Supplementary file 3 [file jcm.00419-26-s0003.pdf]

Tardiomyces depauwii				
Isolate	DNA input conc. (ng/µL)	Number of reads	Genome coverage (%)	Average depth
HMD331004301	2.4	1973990	99.9	104
Lille07.24	1.7	1655616	99.9	127
PSL69.19	3.4	1944377	99.9	113
Tardiomyces blankii				
10-13-09-76	1.9	2160142	99.6	78
10-13-10-05	3.8	1908937	99.7	107
76/2019	3.3	2502103	99.3	85
77/2019	2.4	1727946	99.4	92
78/2019	2.8	1224565	99.5	96
79/2019	3.5	1570535	99.5	84
CBS 1898	2.6	1224053	100	93
CBS 2774	4.3	1540468	99.1	87
CBS 6734	2.7	1945235	99.9	91
CBS 7205	4.2	1803961	99.7	112
Kw593/18	1.8	1971772	99.9	98
NCK2016/106052210	5.2	1809220	99.2	84
NCK2102M17057412	3.6	1611925	98.9	79
NCK230046395903	3.3	2367848	99.3	81
NCK230065787602	2.1	2143317	99.4	86
NCPF8710	4.5	1418414	99.9	94
NCPF8811	3.8	1656852	99.5	101

NCPF8830	3.4	1784599	99.6	97
Rouen002557341	5.9	1505083	99.2	82
Rouen02355627	4.2	1440949	99.1	86
Rouen21735787	2.3	1314385	99.4	94
Rouen803353181	1.8	1642209	99.3	98
Rouen921356552	1.4	1746046	99.2	103
Rouen93836541	3.9	1718268	99.4	87
Rouen949455051	2.7	1517982	99.4	84

Supplemental Data Table S3: Whole genome sequencing metrics of 3 *Tardiomyces depauwii* and 25 *Tardiomyces blankii* isolates.
